# Microbic flow analysis of nano fluid with chemical reaction in microchannel with flexural walls under the effects of thermophoretic diffusion

**DOI:** 10.1038/s41598-023-50915-6

**Published:** 2024-01-17

**Authors:** Noreen Sher Akbar, Maimona Rafiq, Taseer Muhammad, Metib Alghamdi

**Affiliations:** 1 DBS&H, CEME, National University of Sciences and Technology, Islamabad, 44000 Pakistan; 2 Department of Mathematics, COMSATS University Islamabad, Attock, 43600 Pakistan; 3 Department of Mathematics, College of Science, King Khalid University, 61413 Abha, Saudi Arabia

**Keywords:** Biophysics, Mathematics and computing

## Abstract

The current investigation examines the peristaltic flow, in curved conduit, having complaint boundaries for nanofluid. The effects of curvature are taken into account when developing the governing equations for the nano fluid model for curved channels. Nonlinear & coupled differential equations are then simplified by incorporating the long wavelength assumption along with smaller Reynolds number. The homotopy perturbation approach is used to analytically solve the reduced coupled differential equations. The entropy generation can be estimated through examining the contributions of heat and fluid viscosities. The results of velocity, temperature, concentration, entropy number, and stream functions have been plotted graphically in order to discuss the physical attributes of the essential quantities. Increase in fluid velocity within the curved conduit is noticed for higher values of thermophoresis parameter and Brownian motion parameter further entropy generation number is boosted by increasing values of Grashof number.

## Introduction

Biomechanics uses mathematical modelling to investigate medical science-related problems. The movement of bodily fluids within living beings are depicted by biofluid mechanics, a branch of biomechanics. The liquid stream in the blood vessels/respiratory tract/lymphatic and gastro-intestinal systems/urinary tract, and many other physiological systems are tracked and investigated with advanced biofluid mechanics. Recent discoveries are crucial for therapeutic applications include the development of artificial organs, improved vascular vessels, medical equipment design, and material membranes for orthopaedics, among others. Similar bioliquid transport mechanisms may be observed throughout the human body in a variety of contexts. The most prominent of these is peristalsis, which serves as the foundation for the current research. Peristalsis flow was initially discussed and framed by Latham^1^. Shapiro^2^ investigated peristaltic pumping by taking into consideration inertia-free flow in a tube with flexible boundaries by considering small wave number (peristaltic wavelength is larger than tube width). The experimental results Shapiro gave corroborated Latham’s findings. According to existing literature, peristaltic activity within compliant nature boundary geometries have not been yet analysed for various kinds of fluids whether Newtonian/non-Newtonian. The ability of compliant coatings to reduce drag has captivated scientists and engineers. Compliance in physiological systems of humans is the capacity of tubular organs to resist returning to their initial position. The peristaltic flows of Newtonian and non-Newtonian fluids in channels or tubes with compliant limits are the subject of some fascinating studies. Mitra and Prasad^3^ investigated how wall characteristics affected peristaltic motion in a channel. They came to the conclusion that mean flow reversal occurs in the channel's centre and edges. The flow of viscous fluid in thin pipes with elastic walls was explored by Camenschi^4^ and Camenschi and Sandru^5^. A mathematical model was put up by Carew and Pedley^6^ to investigate fluid flow and wall deformation in the human ureter. The stability study of channel flow between compliant boundaries was reported by Davies and Carpenter^7^. Further studies^8–15^ provide an overview of some recent investigations.

Significance of heat transfer cannot be ignored in industrial and medical applications. Research is particularly vital in the domain of heat transport in the human body. Biomedical engineers have become interested in bio-heat transmission in tissues for thermo-therapy^16^ and the thermal- regulation system of humans^17^. Humans transport heat through a variety of mechanisms, including metabolic heat production, arterial oxygenation and venous blood via porous tissue, and tissue conduction. The additional applications include vasodilation, dilution procedure to examine blood flow, and eradication of unwanted cancer tissues. Heat transmission becomes important in oxygenation and hemodialysis in relation to peristalsis. Heat transmission in peristaltically produced flows was the subject of research by several scientists. Mass transfer research is important in relation to heat transfer, particularly in reaction and separation engineering. Drying, energy channeling in damp cooling towers, water surfaces evaporation, and the flow in dessert coolers are just a few examples of the many uses for combined heat and mass transfer. Simultaneous occurrence of heat and mass transfer develops complex relation between the fluxes and the driving potentials. Energy flux due to concentration gradient is termed as thermal diffusion or Dufour effect whereas mass flux geneerated by temperature gradient is known as Soret effect. Although mass diffusion through heat and heat diffusion via concentration gradient are viewed as being of a lower order of magnitude when compared to influences resulting from Fouriers or Ficks law, there are circumstances in which such effects cannot be disregarded. For instance, the thermal diffusion phenomenon is used for isotope separation, and the diffusion-thermo effect needs to be present in mixtures of gases with high molecular weight (H_2_, He) and medium molecular weight (H_2_, air)^18^. Furthermore, when nutrients diffuse from blood arteries to the surrounding tissues, blood flow throughout the human body simultaneously involves heat/mass transference. Ogulu^19^ analyzed the heat production effects on the fluid flow with small Reynolds number and mass transport in a lymphatic vessel under the influence of uniform magnetic field. Entropy, which is frequently interpreted as a measure of disorder or of the movement towards thermodynamic equilibrium, can be taken as distinct number of ways a thermodynamic system may be arranged in thermodynamics. Pakdemirli and Yilbas^20^ address non-Newtonian liquid flow via pipe system with entropy generation. They propose that as the Brinkman number grows, the entropy number does as well. The rate of entropy formation for a peristaltic pump was discussed by Souidi et al.^21^. You can examine a few current articles on the subject by using the references^22–33^.

Entropy generation for peristaltic flow in a curved channel with complaint walls is not yet inspected so far. Therefore, to fill the void, we have presented the entropy generation analysis via curved conduit with complaint walls with peristaltic activity because thermodynamic analysis of entropy generation in peristaltic fow through a curved channel with compliant walls has applications in biomedical devices, Implications for energy efficiency in microfluidic systems, Applications in drug delivery system, Lab-on-a-chip devices, Implications for energy efficiency in industrial pumps etc**.** The lubrication assumption is integrated in the flow equations. The reduced coupled differential equations are solved analytically with the help of homotopy perturbation method. The entropy generation is computed by evaluation of thermal and fluid viscosities contribution. The physical features of pertinent parameters have been discussed by plotting the graphs of velocity, temperature, concentration, entropy number and stream functions.

## Mathematical formulation

**Figure 1:**
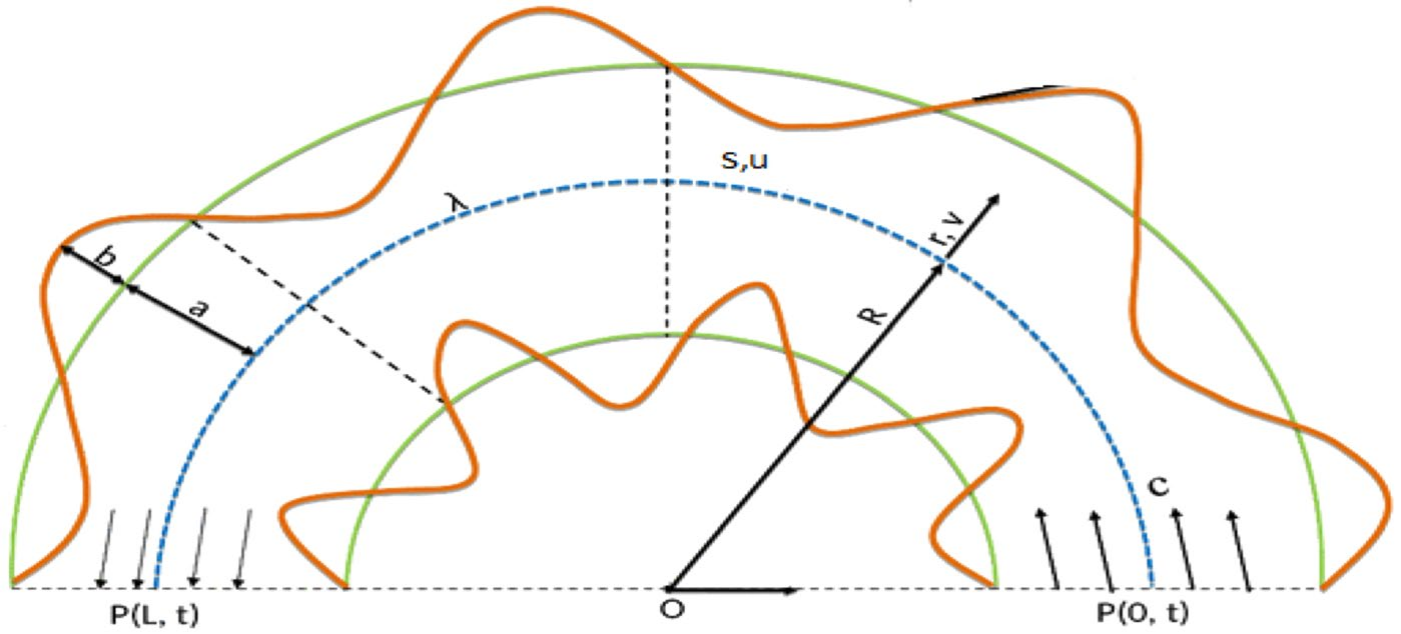
Geometry of the problem.

The flow of an incompressible nanofluid in two dimensions through a curved conduit with uniform thickness $2a$ is taken into consideration. The flexible channel walls can be compared to a complaint nature that is subjected to the imposition of a travelling wave with lower amplitudes. Defining $R^{*}$ as the radius of curvature and $\left( {\overline{N}, \overline{S}, \overline{Z}} \right)$ as the respective cross-stream, downstream, and perpendicular directions, the course of flow through conduit is originated via small amplitude $b$ oscillating waves moving on the edge of the bendable walls of conduit see Fig. 1. The walls geometry mathematical expression is as follows:

1 $$H\left( {\overline{S},\overline{t}} \right) = a + b\sin \left[ {\frac{2\pi }{\lambda }\left( {\overline{S} - c\overline{t}} \right)} \right],\;\;{\text{upper wall}}$$

2 $$- H\left( {\overline{S},\overline{t}} \right) = - a - b\sin \left[ {\frac{2\pi }{\lambda }\left( {\overline{S} - c\overline{t}} \right)} \right].\;\;{\text{lower}}\;{\text{wall}}$$

The wave speed and wave length are indicated in the equations above by the letter $c$ and $\lambda$, respectively. Let $\overline{V}$/$\overline{U}$ stand for the cross and down-stream velocity components, respectively. The following equations described the motion, energy, and nanoparticle volume fraction for curved channels^26^.

3 $$\frac{{R^{*} }}{{\overline{N} + R^{*} }}\frac{\partial }{{\partial \overline{N}}}\left( {\frac{{\left( {\overline{N} + R^{*} } \right)}}{{R^{*} }}\overline{V}} \right) + \frac{{R^{*} }}{{\overline{N} + R^{*} }}\frac{{\partial \overline{U}}}{{\partial \overline{S}}} = 0,$$

4 $$\begin{aligned} \rho_{f} & \left( {\frac{{\partial \overline{V}}}{{\partial \overline{t}}} + \overline{V}\frac{{\partial \overline{V}}}{{\partial \overline{N}}} + \frac{{R^{*} \overline{U}}}{{\overline{N} + R^{*} }}\frac{{\partial \overline{V}}}{{\partial \overline{S}}} - \frac{{\overline{U}^{2} }}{{\overline{N} + R^{*} }}} \right) \\ & = - \frac{{\partial \overline{P}}}{{\partial \overline{N}}} + \frac{\partial }{{\partial \overline{N}}}\left( {\overline{\tau }_{{\overline{N}\overline{N}}} } \right) + \frac{{R^{*} }}{{\overline{N} + R^{*} }}\frac{\partial }{{\partial \overline{S}}}\left( {\overline{\tau }_{{\overline{N}\overline{S}}} } \right) - \frac{{\overline{\tau }_{{\overline{S}\overline{S}}} }}{{\overline{N} + R^{*} }}, \\ \end{aligned}$$

5 $$\begin{aligned} \rho_{f} & \left( {\frac{{\partial \overline{U}}}{{\partial \overline{t}}} + \overline{V}\frac{{\partial \overline{U}}}{{\partial \overline{N}}} + \frac{{R^{*} \overline{U}}}{{\overline{N} + R^{*} }}\frac{{\partial \overline{U}}}{{\partial \overline{S}}} + \frac{{\overline{U}\overline{V}}}{{\overline{N} + R^{*} }}} \right) \\ & = - \frac{{R^{*} }}{{\overline{N} + R^{*} }}\frac{{\partial \overline{P}}}{{\partial \overline{S}}} + \frac{\partial }{{\partial \overline{N}}}\left( {\overline{\tau }_{{\overline{N}\overline{S}}} } \right) + \frac{{R^{*} }}{{\overline{N} + R^{*} }}\frac{\partial }{{\partial \overline{S}}}\left( {\overline{\tau }_{{\overline{S}\overline{S}}} } \right) \\ & \;\; + \rho g\alpha \left( {\overline{T} - T_{0} } \right) + \rho g\alpha \left( {\overline{C} - C_{0} } \right), \\ \end{aligned}$$

6 $$\left( {\rho c} \right)_{f} \frac{{d\overline{T}}}{{d\overline{t}}} = \kappa \nabla^{2} \overline{T} + \left( {\rho c} \right)_{p} \left[ {D_{B} \nabla \overline{C} \cdot \nabla \overline{T} + \frac{{D_{T} }}{{T_{0} }}\nabla \overline{T} \cdot \nabla \overline{T}} \right] + \overline{\tau }.\overline{L},$$

7 $$\frac{{d\overline{C}}}{{d\overline{t}}} = D_{B} \nabla^{2} \overline{C} + \frac{{D_{T} }}{{T_{0} }}\nabla^{2} \overline{T}.$$

In the above equations, the symbol $\overline{P}$ stands for pressure while $\overline{V}$/$\overline{U}$ depict velocity elements in cross ($\overline{N}$)/down ($\overline{S}$) stream directions respectively. The following are the defined boundary conditions for a symmetric channel with compliant walls:

8 $$\overline{U} = 0{\text{ at }}\overline{N} = \pm H = \pm \left[ {a + b\sin \left( {\frac{2\pi }{\lambda }\left( {\overline{S} - c\overline{t}} \right)} \right)} \right],$$

9 $$\frac{{R^{*} }}{{\overline{N} + R^{*} }}\frac{\partial }{{\partial \overline{S}}}L\left( H \right) = \frac{{R^{*} }}{{\overline{N} + R^{*} }}\frac{{\partial \overline{P}}}{{\partial \overline{S}}}{\text{ at }}\overline{N} = \pm H,$$

10 $$\overline{T} = T_{0} ,\overline{C} = C_{0} {\text{ on }}\overline{N} = - H,$$

11 $$\overline{T} = T_{1} ,\overline{C} = C_{1} {\text{ on }}\overline{N} = H.$$

Here fluid density is shown via $\rho_{f}$, viscosity through $\mu_{f}$ and $\left( {\rho c} \right)_{f}$ shows heat capacitance. In addition, $\left( {\rho c} \right)_{p}$ is the nano-particle heat capacity, $k$ defines thermal conductance ability whereas $D_{B}$ and $D_{T}$ are Brownian motion/thermophoretic coefficients respectively. Moreover,

12 $$L = B\frac{{\partial^{4} }}{{\partial \overline{S}^{4} }} - \sigma \frac{{\partial^{2} }}{{\partial \overline{S}^{2} }} + m\frac{{\partial^{2} }}{{\partial t^{2} }} + C\frac{\partial }{\partial t} + K,$$

where $B$ and portray flexural rigidity and longitudinal temsion/width repectively whereas $m, C$ and $K$ show the mass/Area, viscous damping coefficient and spring stiffness respectively. Introducing the link between the velocity stream function and the following non-dimensional variables.

13 $$\begin{aligned} s & = \frac{{\overline{S}}}{\lambda },n = \frac{{\overline{N}}}{a},u = \frac{{\overline{U}}}{c},v = \frac{{\overline{V}}}{c},t = \frac{{c\overline{t}}}{\lambda },k = \frac{{R^{*} }}{a},{\text{Re}} = \frac{{\rho_{f} ca}}{\mu },\varepsilon = \frac{b}{a},\alpha = \frac{\kappa }{{\left( {\rho c} \right)_{f} }}, \\ \delta & = \frac{a}{\lambda },P = \frac{{a^{2} \overline{P}}}{c\mu \lambda },\theta = \frac{{\overline{T} - T_{1} }}{{T_{0} - T_{1} }},\sigma = \frac{{\overline{C} - C_{1} }}{{C_{0} - C_{1} }},G_{r} = \frac{{g\alpha a^{2} \left( {T_{0} - T_{1} } \right)}}{\nu c},\gamma = \frac{{\mu c^{2} }}{{\left( {T_{1} - T_{0} } \right)\alpha \left( {\rho c} \right)_{f} }}, \\ E_{4} & = - \frac{{\sigma a^{3} }}{{\lambda^{3} \mu c}},E_{1} = \frac{{mca^{3} }}{{\lambda^{3} \mu }},E_{2} = \frac{{Ca^{3} }}{{\lambda^{2} \mu }},E_{3} = \frac{{Ba^{3} }}{{\mu c\lambda^{5} }},E_{5} = \frac{{Ka^{3} }}{\mu c\lambda },B_{r} = \frac{{g\alpha a^{2} \left( {C_{0} - C_{1} } \right)}}{\nu c}, \\ N_{b} & = \frac{{\left( {\rho c} \right)_{p} D_{B} C_{0} }}{{\alpha \left( {\rho c} \right)_{f} }},N_{t} = \frac{{\left( {\rho c} \right)_{p} D_{T} T_{0} }}{{\alpha \left( {\rho c} \right)_{f} }},u = - \frac{\partial \psi }{{\partial n}},v = \delta \frac{k}{n + k}\frac{\partial \psi }{{\partial s}}. \\ \end{aligned}$$

Equation (3) is identically satisfied. Invoking lubrication assumption into Eqs. (4–11), following dimensionless form is obtained.

14 $$\frac{\partial P}{{\partial n}} = 0,$$

15 $$\left( {\frac{k}{n + k}} \right)\frac{\partial P}{{\partial s}} = \frac{\partial }{\partial n}\left( {\frac{\partial u}{{\partial n}} - \frac{u}{n + k}} \right) + G_{r} \theta + B_{r} \sigma ,$$

16 $$\frac{{\partial^{2} \theta }}{{\partial n^{2} }} + \frac{1}{n + k}\frac{\partial \theta }{{\partial n}} + N_{b} \frac{\partial \sigma }{{\partial n}}\frac{\partial \theta }{{\partial n}} + N_{t} \left( {\frac{\partial \theta }{{\partial n}}} \right)^{2} + 2\gamma \frac{\partial u}{{\partial n}}\left( {\frac{\partial u}{{\partial n}} - \frac{u}{n + k}} \right) = 0,$$

17 $$\frac{\partial }{\partial n}\left[ {\left( {n + k} \right)\frac{\partial \sigma }{{\partial n}}} \right] + \frac{{N_{t} }}{{N_{b} }}\frac{\partial }{\partial n}\left[ {\left( {n + k} \right)\frac{\partial \theta }{{\partial n}}} \right] = 0,$$

18 $$u = 0,{\text{ at }}n = \pm h = \pm \left[ {1 + \varepsilon \sin \left( {s - t} \right)} \right],$$

19 $$\left( {\frac{k}{n + k}} \right)\frac{\partial P}{{\partial s}} = \left[ {E_{1} \frac{{\partial^{3} h}}{{\partial t^{2} \partial s}} + E_{2} \frac{{\partial^{2} h}}{\partial t\partial s} + E_{3} \frac{{\partial^{5} h}}{{\partial s^{5} }} + E_{4} \frac{{\partial^{3} h}}{{\partial s^{3} }} + E_{5} \frac{\partial h}{{\partial s}}} \right]{\text{ at }}n = \pm h,$$

20 $$\theta = 1,\sigma = 1{\text{ at }}n = - h,$$

21 $$\theta = 0,\sigma = 0{\text{ at }}n = h.$$

## Mathematical solution

We create the following homotopy equations for linked nonlinear differential Eqs. (14–21) to be solved by HPM.

22 $$\begin{aligned} H\left( {u,p} \right) & = \left( {1 - p} \right)\left[ {\frac{{\partial^{2} u}}{{\partial n^{2} }} - \frac{{\partial^{2} u_{01} }}{{\partial n^{2} }}} \right] \\ & \;\;\; + p\left[ {\frac{\partial }{\partial n}\left( {\frac{\partial u}{{\partial n}} - \frac{u}{n + k}} \right) + G_{r} \theta + B_{r} \sigma - \frac{k}{{\left( {n + k} \right)}}\frac{\partial P}{{\partial s}}} \right] = 0, \\ \end{aligned}$$

23 $$\begin{aligned} H\left( {\theta ,p} \right) & = \left( {1 - p} \right)\left[ {\left( {\frac{{\partial^{2} \theta }}{{\partial n^{2} }} + \frac{1}{n + k}\frac{\partial \theta }{{\partial n}}} \right) - \left( {\frac{{\partial^{2} \theta_{01} }}{{\partial n^{2} }} + \frac{1}{n + k}\frac{{\partial \theta_{01} }}{\partial n}} \right)} \right] \\ & \;\;\; + p\left[ {\frac{{\partial^{2} \theta }}{{\partial n^{2} }} + \frac{1}{n + k}\frac{\partial \theta }{{\partial n}} + N_{b} \frac{\partial \sigma }{{\partial n}}\frac{\partial \theta }{{\partial n}} + N_{t} \left( {\frac{\partial \theta }{{\partial n}}} \right)^{2} + \gamma \frac{\partial u}{{\partial n}}\left( {\frac{\partial u}{{\partial n}} - \frac{u}{n + k}} \right)} \right] = 0, \\ \end{aligned}$$

24 $$\begin{aligned} H\left( {\sigma ,p} \right) & = \left( {1 - p} \right)\left[ {\frac{{\partial^{2} \sigma }}{{\partial n^{2} }} + \frac{1}{n + k}\frac{\partial \sigma }{{\partial n}} - \left( {\frac{{\partial^{2} \sigma_{01} }}{{\partial n^{2} }} + \frac{1}{n + k}\frac{{\partial \sigma_{01} }}{\partial n}} \right)} \right] \\ & \;\;\; + p\left[ {\frac{{\partial^{2} \sigma }}{{\partial n^{2} }} + \frac{1}{n + k}\frac{\partial \sigma }{{\partial n}} + \frac{{N_{t} }}{{N_{b} }}\left( {\frac{{\partial^{2} \theta }}{{\partial n^{2} }} + \frac{1}{n + k}\frac{\partial \theta }{{\partial n}}} \right)} \right] = 0. \\ \end{aligned}$$

Using

25 $$u = u_{0} + pu_{1} + p^{2} u_{2} + \cdots ,$$

26 $$\theta = \theta_{0} + p\theta_{1} + p^{2} \theta_{2} + \cdots ,$$

27 $$\sigma = \sigma_{0} + p\sigma_{1} + p^{2} \sigma_{2} + \cdots$$

Incorporation of Eqs. (25–27) into Eqs. (22–24) along with considered boundary conditions (18–21), the solutions can be directly written as when p → 1. The drawn out solutions are graphically discussed in next section in detail. The following formula, developed by Pakdemirli and Yilbas^20^, has been used to calculate the expression for the EGN (i.e. entropy generation number):

28 $$N_{s} = \left( {\frac{\partial \theta }{{\partial n}}} \right)^{2} + 2\gamma \theta_{0} \frac{\partial u}{{\partial n}}\left( {\frac{\partial u}{{\partial n}} - \frac{u}{n + k}} \right).$$

In (28), the Ist term on the R.H.S. of Eq. is the result of heat generation and can be presented as $N_{S1}$, and the $2{\text{nd}}$ term shows viscous dissipation as $N_{S2}$, i.e.

29 $$N_{{s_{1} }} = \left( {\frac{\partial \theta }{{\partial n}}} \right)^{2} ,$$

30 $$N_{{s_{2} }} = 2\gamma \theta_{0} \frac{\partial u}{{\partial n}}\left( {\frac{\partial u}{{\partial n}} - \frac{u}{n + k}} \right).$$

## Result analysis

**Figure 2:**
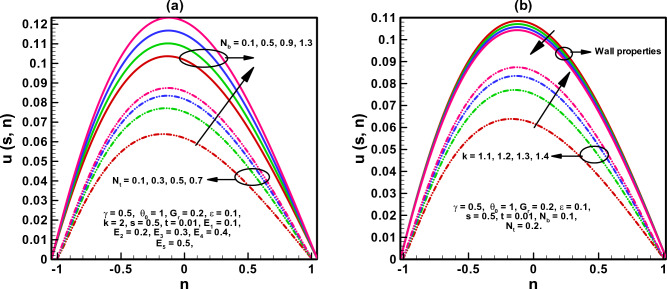
(**a**, **b**) Velocity profile.

**Figure 3:**
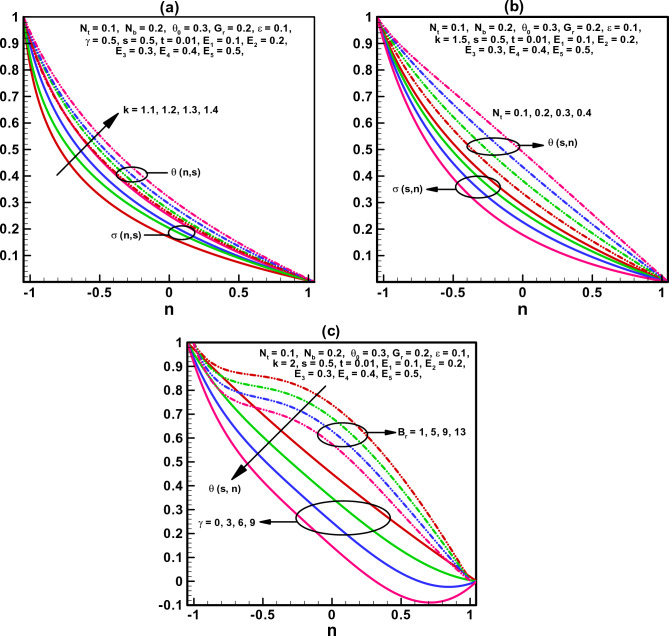
(**a**–**c**) Temperature and concentration profile.

**Figure 4:**
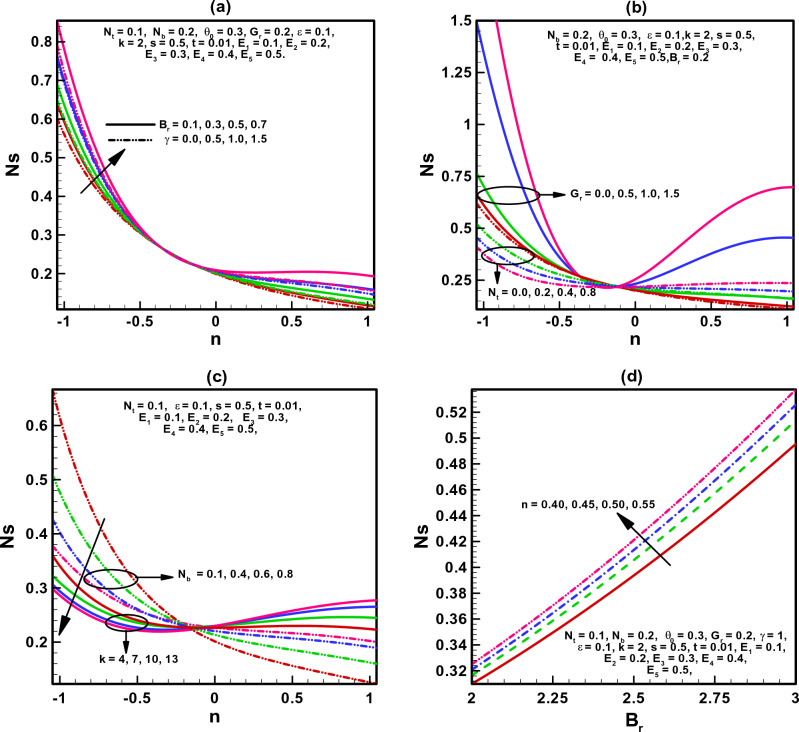
(**a**–**d**) Variation of entropy generation number N_S_.

**Figure 5:**
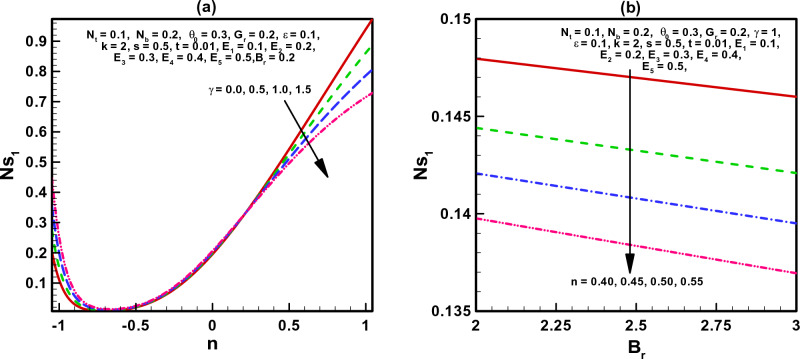
(**a**, **b**) Variation of heat generation Ns_1_.

**Figure 6:**
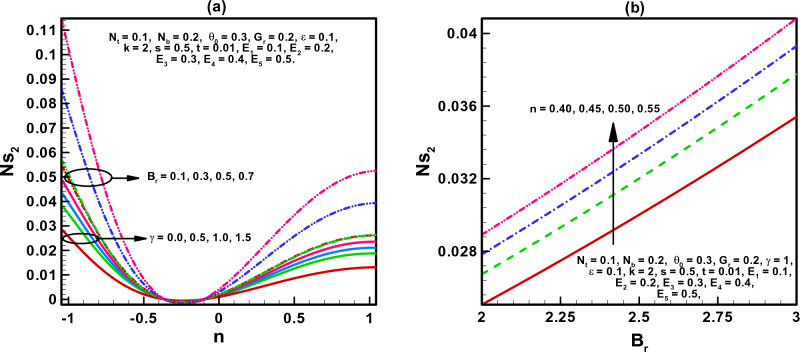
(**a**, **b**) Variation of Ns_2_.

**Figure 7:**
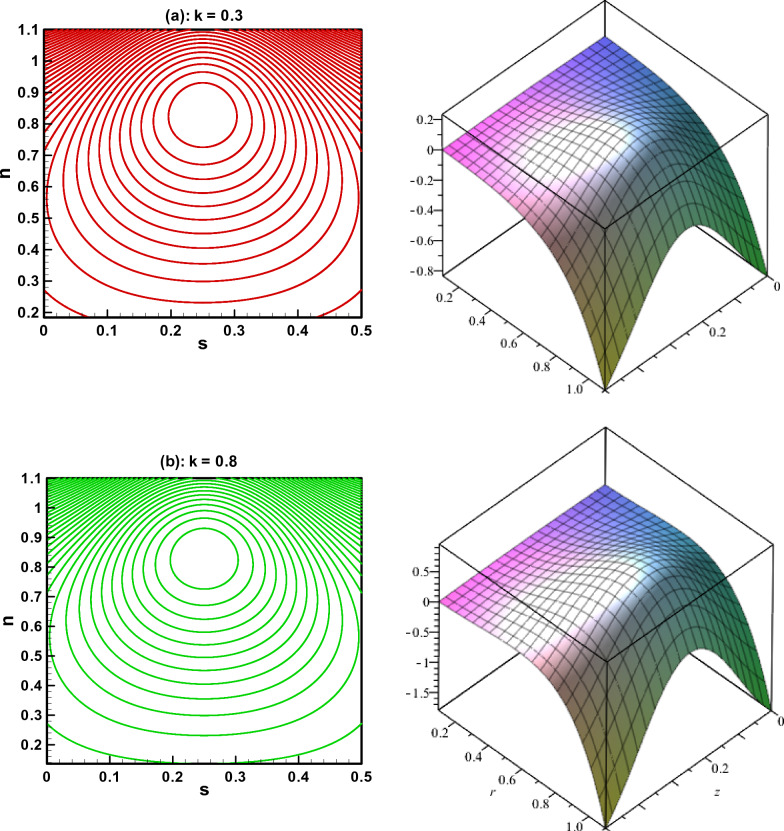
(**a**, **b**) Streamlines for different of $k$.

**Figure 8:**
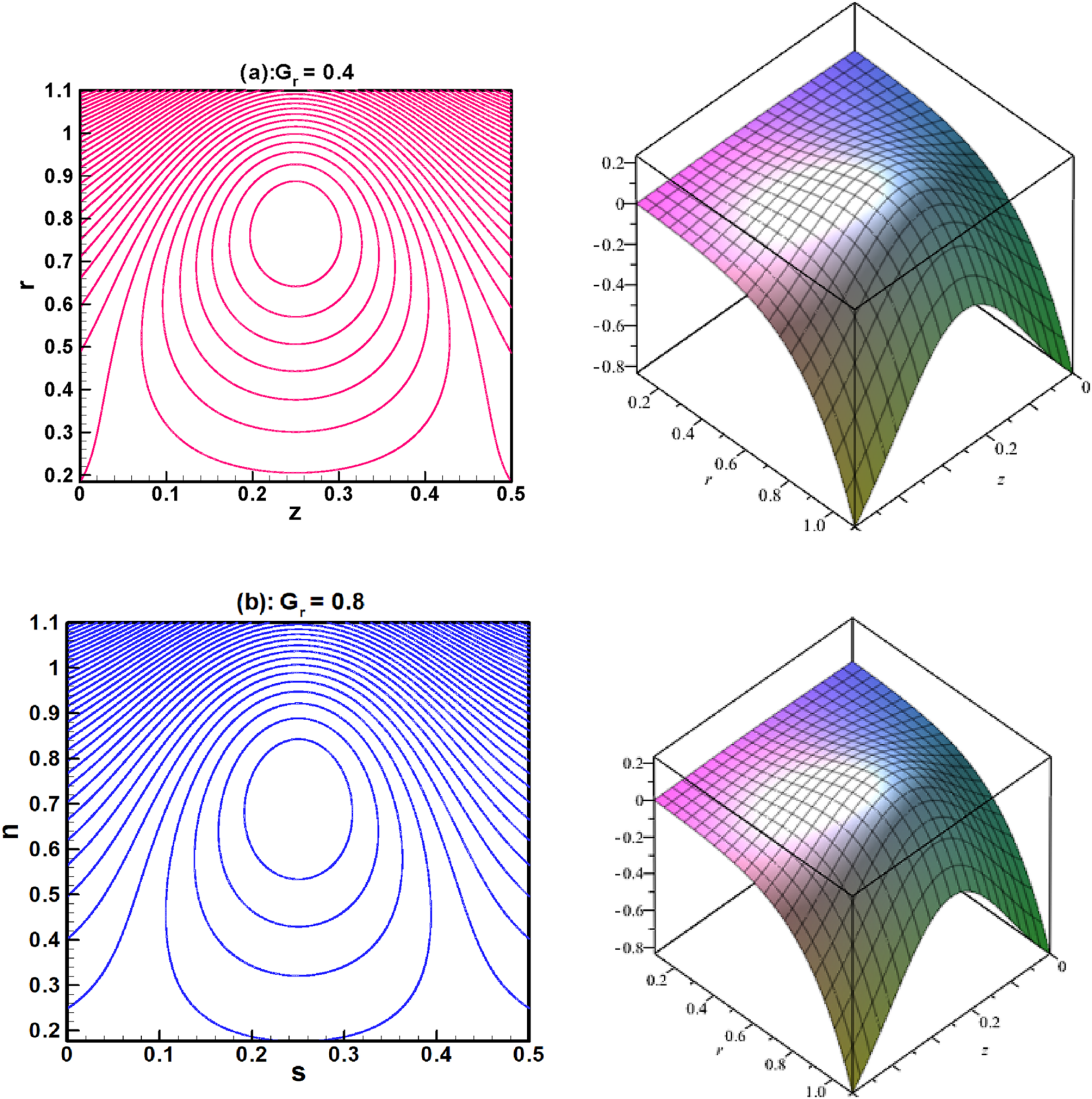
(**a**, **b**) Streamlines for different of $G_{r}$.

The visual findings of significant parameters for velocity, temperature, concentration, entropy, heat generation, and viscous dissipation are discussed in this section. Figure 2a, b are the graphs for fluid velocity. It is observed that the fluid velocity enhances within the curved channel by raising the values of $N_{b}$ and $N_{t}$ respectively. Moreover, the channel curvature and complaint wall properties play vital role in the reduction of fluid speed within the curved channel. For all the parameters fluid attains maximum velocity in the middle region of the curved channel. Figure 3a–c displays the plots for temperature and concentration profiles. It is noted that the increase in curve-ness of the channel raise the temperature and concentration of nano particles suspended in the fluid. The increase in thermophoresis parameter $N_{t}$ contributes in decreasing the concentration of nano particles while increasing the temperature profile. Figure 3c shows the effect of local nano particle Grashof number $G_{r}$ and heat dissipation parameter $\gamma$. The raise in values of both parameters reduces nano particle temperature respectively. Figure 4a–d are graphical results for entropy generation number $N_{S}$. These graphical results indicate that increasing values of $B_{r}$, $G_{r}$ and $\gamma$ boost entropy generation number. The rasing values of $N_{t}$, $N_{b}$ and $k$ contributes in reducing entropy generation number in the inner half of the channel whereas near the outer half of the channel entropy generation number raises with increasing values of these parameters respectively. Moreover, $N_{S}$ is plotted against $B_{r}$ in Fig. 4d to study $N_{S}$ behavior at different radial distances within the channel. It is concluded that the entropy generation number increases as radial distance increases in the central region of the curved channel. Heat generation $N_{S1}$ effect are shown in Fig. 5a, b. It is seen that the increase in heat dissipation parameter $\gamma$, minimize the heat generation. Also in the middle section of the curved channel, heat generation decreases via enhancing radial distance. Viscous dissipation effect are plotted in Fig. 6a, b. Heat dissipation parameter $\gamma$ as well as $B_{r}$ contributes in reducing viscous dissipation respectively. It is also noticed that in the central region of the curved channel viscous dissipation raises with increasing radial distance. Streamlines are plotted for different values of curvature parameter $k$ in Fig. 7a, b. It is observed that the increase in $k$ increases number of closed streamlines. Additionally, the trapping bolus is visible in the channel's outermost section. The streamlines for various values of $G_{r}$ are shown in Fig. 8a, b. It is noted that the increase in value of $G_{r}$ changes the orientation of the trapping bolus i.e. from round to oval. Moreover, number of closed streamlines reduces with increasing values of $G_{r}$. Table 1 gives numerical values of velocity, temperature, and concentration profile for different values of flow parameters. Table 2 gives numerical values of entropy generation for different flow parameters.

**Table 1:** Numerical values of velocity, temperature and concentration profile for different values $k$ and $N_{t}$ when: $N_{b} = 0.2$, $G_{r} = 0.3,$ $\gamma = 0.5,$ $\theta_{0} = 1,$ $\varepsilon = 0.1,$ $B_{r} = 0.2,$ $s = 0.5,$ $t = 0.01,$ $E_{1} = 0.1,$ $E_{2} = 0.2,$ $E_{3} = 0.3,$ $E_{4} = 0.4,$ $E_{5} = 0.5.$

*n*	*k*	*u(n, s)*	*θ(n, s)*	*C(n, s)*	*n*	*N*_*t*_	*u(n, s)*	*θ(n, s)*	*C(n, s)*
− 1.0	1.1	0.000	1.000	1.000	− 1.0	0.1	0.000	1.000	1.000
− 0.4	–	0.059	0.354	0.305	− 0.4	–	0.092	0.545	0.492
0.4	–	0.038	0.099	0.081	0.4	–	0.071	0.170	0.143
1.0	–	0.000	0.000	0.000	1.0	–	0.000	0.000	0.000
–	1.2	0.000	1.000	1.000	–	0.4	0.000	1.000	1.000
–	–	0.070	0.442	0.388	–	–	0.108	0.693	0.361
–	–	0.047	0.129	0.106	–	–	0.086	0.278	0.075
–	–	0.000	0.000	0.000	–	–	0.000	0.000	0.000

**Table 2:** Numerical values of entropy generation number for different values $k$ when: $N_{b} = 0.2$, $G_{r} = 0.3,$ $\gamma = 0.5,$ $\theta_{0} = 1,$ $\varepsilon = 0.1,$ $B_{r} = 0.2,$ $s = 0.5,$ $t = 0.01,$ $E_{1} = 0.1,$ $E_{2} = 0.2,$ $E_{3} = 0.3,$ $E_{4} = 0.4,$ $E_{5} = 0.5.$

*n*	*k*	*N*_*s*_	$N_{{s_{1} }}$	$N_{{s_{2} }}$	*n*	*k*	*N*_*s*_	$N_{{s_{1} }}$	$N_{{s_{2} }}$
− 1.0	4	0.377	0.377	0.119	− 1.0	10	0.306	0.325	0.148
− 0.4	–	0.253	0.012	0.008	− 0.4	–	0.224	0.007	0.01
0.4	–	0.217	0.476	0.028	0.4	–	0.250	0.454	0.028
1.0	–	0.210	0.815	0.043	1.0	–	0.261	0.950	0.045
–	7	0.322	0.338	0.142	–	13	0.298	0.319	0.151
–	–	0.232	0.008	0.012	–	–	0.220	0.007	0.014
–	–	0.241	0.463	0.028	–	–	0.255	0.449	0.027
–	–	0.245	0.912	0.045	–	–	0.269	0.970	0.045

## Conclusion

The current research investigates about the peristaltic flow of nano-liquid through curved flexiable channel in the presence of Brownian motion and thermophoresis. Main findings of the study are listed below:

- Increase in fluid velocity within the curved conduit is noticed for higher values of $N_{b}$ and $N_{t}$ respectively.
- Entropy generation number is boosted by increasing values of $B_{r}$, $G_{r}$ and $\gamma$.
- Streamlines get closed as we increase the curvature of channel.

## Data Availability

The datasets used and/or analyzed during the current study available from the corresponding author on reasonable request.

## References

[1] Latham, T. W. Fluid motion in a peristaltic pump, MS. Thesis, Massachusetts Institute of Technology, Cambridge (1966).

[2] Shapiro, A. H. Pumping and retrograde diffusion in peristaltic waves. In *Proceedings of the Workshop on Ureteral Reflux in Children. Nat. Acad. Sci., Washington, D. C.* 109–126 (1967).

[3] Mitra TK, Prasad SN (1973). On the influence of wall properties and Poiseuille flow in peristalsis. J. Biomech..

[4] Camenschi G (1977). The motion of a Newtonian viscous fluid through thin pipe with thin linear elastic wall. Lett. Appl. Eng. Sci..

[5] Camenschi G, Sandru N (1979). A model of a viscous fluid motion through an axisymmetrical deformable pipe with thin linear elastic wall. Roum. Math. Pures Et Appl..

[6] Carew EO, Pedley TJ (1997). An active membrane model for peristaltic pumping: Part 1—periodic activation waves in an infinite tube. Trans. ASME J. Biomech. Eng..

[7] Davies C, Carpenter PW (1997). Instabilities in a plane channel flow between compliant walls. J. Fluid Mech..

[8] Sankad GC, Nagathan PS (2016). Unsteady MHD peristaltic flow of a couple stress fluid through porous medium with wall and slip effects. Alex. Eng. J..

[9] Akbar S, Sohial M (2022). Three dimensional MHD viscous flow under the influence of thermal radiation and viscous dissipation. Int. J. Emerg. Multidiscipl. Math..

[10] Li S, Akbar S, Sohail M, Nazir U, Singh A, Alanazi M, Hassan AM (2023). Influence of buoyancy and viscous dissipation effects on 3D magneto hydrodynamic viscous hybrid nano fluid (MgO− TiO2) under slip conditions. Case Stud. Therm. Eng..

[11] Selvi CK, Srinivas ANS (2019). Peristaltic transport of Herschel-Bulkley fluid in a non-uniform elastic tube. Propuls. Power Res..

[12] Yasmeen S, Asghar S, Anjum HJ, Ehsan T (2019). Analysis of Hartmann boundary layer peristaltic flow of Jeffrey fluid: Quantitative and qualitative approaches. Commun. Nonlinear Sci. Numer. Simul..

[13] Ali A, Awais M, Al-zubaidi A, Saleem S, Khan-Marwat DN (2022). Hartman boundary layer peristaltic flow for viscoelastic fluid. Ain Shams Eng. J..

[14] Ahmed B, Ashraf A, Anwar F (2023). Inertial considerations in peristaltically activated MHD blood flow model in an asymmetric channel using Galerkin finite element simulation for moderate Reynolds number. Alex. Eng. J..

[15] Priyadarsini GD, Sankad GC (2023). Wall consequences for the peristaltic movement of non-Newtonian fluid in an inclined conduit. Mater. Today: Proc..

[16] Shera MD, Gladman AS, Davidson SR, Trachtenberg J, Gertner MR (2001). Helical antenna arrays for interstitial microwave thermal therapy for prostate cancer: Tissue phantom testing and simulations for treatment. Phys. Med. Biol..

[17] Sanyal DC, Maji NK (2001). Thermoregulation through skin under variable atmospheric and physiological conditions. J. Theor. Biol..

[18] Gupta PS, Gupta AS (1977). Heat and mass transfer on stretching sheet with suction or blowing. Can. J. Chem. Eng..

[19] Ogulu A (2006). Effect of heat generation on low Reynolds number fluid and mass transport in a single lymphatic blood vessel with uniform magnetic field. Int. Commun. Heat Mass Transfer.

[20] Pakdemirli M, Yilbas BS (2006). Entropy generation in a pipe due to non-Newtonian fluid flow: Constant viscosity case. Sadhana.

[21] Souidi F, Ayachi K, Benyahia N (2009). Entropy generation rate for a peristaltic pump. J. Non-Equilib. Thermodyn..

[22] Noreen S, Kousar T (2019). Hall, ion slip and ohmic heating effects in thermally active sinusoidal channel. Propuls. Power Res..

[23] Zidan AM, McCash LB, Akhtar S, Saleem A, Issakhov A, Nadeem S (2021). Entropy generation for the blood flow in an artery with multiple stenosis having a catheter. Alex. Eng. J..

[24] Sharma BK, Gandhi R, Bhatti MM (2022). Entropy analysis of thermally radiating MHD slip flow of hybrid nanoparticles (Au-Al_2_O_3_/Blood) through a tapered multi-stenosed artery. Chem. Phys. Lett..

[25] Patil PM, Shankar HF (2022). Heat transfer attributes of Al_2_O_3_-Fe_3_O_4_/H_2_O hybrid nanofluid flow over a yawed cylinder. Propuls. Power Res..

[26] Akram J, Akbar NS, Alansari M, Tripathi D (2022). Electroosmotically modulated peristaltic propulsion of TiO_2_/10W40 nanofluid in curved microchannel. Int. Commun. Heat Mass Transfer.

[27] Akram J, Akbar NS (2022). Entropy generation in electroosmotically aided peristaltic pumping of MoS_2_ Rabinowitsch nanofluid. Fluid Dyn. Res..

[28] Rehman S, Hashim F, Al-Yarimi AM, Alqahtani S, Awad M (2023). Dissipative flow features of Carreau nanofluid with thermal radiation inside plane wall channel: Jeffery-Hamel analysis. Propuls. Power Res..

[29] Bhatti MM, Sara I (2022). Abdelsalam, scientific breakdown of a ferromagnetic nanofluid in hemodynamics: Enhanced therapeutic approach. Math. Model. Nat. Phenom..

[30] Nazir U, Sohail M, Mukdasai K, Singh A, Alahmadi RA, Galal AM, Eldin SM (2022). Applications of variable thermal properties in Carreau material with ion slip and Hall forces towards cone using a non-Fourier approach via FE-method and mesh-free study. Front. Mater..

[31] Imran N, Javed M, Sohail M, Qayyum M, Khan RM (2023). Multi-objective study using entropy generation for Ellis fluid with slip conditions in a flexible channel. Int. J. Modern Phys. B.

[32] Maraj EN, Akbar NS, Zehra I, Butt AW, Ahmed-Alghamdi H (2023). Electro-osmotically modulated magneto hydrodynamic peristaltic flow of menthol based nanofluid in a uniform channel with shape factor. J. Magn. Magn. Mater..

[33] Bhatti MM, Ishtiaq F, Ellahi R, Sait SM (2023). Novel aspects of cilia-driven flow of viscoelastic fluid through a non-Darcy medium under the influence of an induced magnetic field and heat transfer. Mathematics.

